# Ovariotomy Successfully Performed by Dr. John Craig, of Stanford, Kentucky

**Published:** 1855-08

**Authors:** T. F. Craig

**Affiliations:** Stanford


### ART. IV.—OVARIOTOMY SUCCESSFULLY PERFORMED BY DR. JOHN CRAIG, OF STANFORD, KENTUCKY.

REPORTED BY DR. T. F. CRAIG.

Mrs. H., aged 26, a native of Kentucky, menstruated in her fifteenth year, married at twenty, gave birth to her only child six years later; from her eight to her twentieth year had been afflicted with otorrhœa and enlarged cervical glands; at sixteen caught a bad cold, which resulted in suppression of the menses. This lasted tenth months, when her health improved, although menstruation never became regular again. When first seen by Dr. Craig, (April 8th, 1854,) she had been, since her confinement, under the care of Dr. Pierce, of Lancaster, Ky., a period of ten months. This gentleman pronounced the case to be one of ovarian tumor. At the period of Pierce's first visit, shortly after her confinement, the outline of the tumor was indistinctly defined, on account of the coexistence of ascites. This was, however, readily removed by treatment; and the tumor, firm, irregular and very moveable, found lying in the left iliac region. The patient complained of a dull aching pain in the region of the tumor, at the time of her first menstruatien following her confinement, (her child lived but a short time), and this continued up to the time of the operation. Effusion into the peritoneal cavity occurred two or three times, in the period between her accouchment and the removal of the tumor. This, however, disappeared readily, under appropriate treatment. Previous to the confinement of the patient no suspicion of the existence of the tumor had been entertained, but she was remarkably large, and was thought by her friends to be pregnant with twins. At the time of Dr. Craig's first visit there existed a considerable effusion into the abdominal cavity and the tumor could be only indistinctly defined. She was put upon the use of acet. of potassa, which by the next visit (April 13,) had caused the effusion to disappear. The tumor felt to the hand firm, irregular and slightly moveable. It extended from the pubis to the epigastrium, filling up the left side, and a portion of the right. Adherent ovarian tumor was the diagnosis made. The patient was informed of the nature of her disease and of the necessity of an operation for its cure, the dangers of which, as well as that of allowing the tumor to remain, being explained to her she decided to undergo the operation. A consultation was held on the 17th, at which time the patient seemed very desirous of having the operation performed. This was accordingly fixed for the 22d. On this day, at 12 o'clock, Drs. Pierce, Cook, Jackson and myself being present, the patient was placed upon the table. I was entrusted with the administration of the sulph. ether. Her pulse was slow and feeble, her skin cool and moist, and she seemed quite composed, the circumstances being considered. The operation was begun by Dr. Craig, Drs. Cook and Peirce assisting.

An explorative incision was made three inches long, between the pubis and umbilicus; after carefully dissecting down to the peritoneum, this was opened, giving exit to about twenty ounces of serum, and leaving exposed the firm, irregular, silvery looking tumor. The exploring finger could now detect adhesion of the tumor to the peritoneum in the left lumbar region, no other being found after a careful examination, it was decided to extend the incision to the pubis and umbilicus. At this time about a pint of serum again escaped. A large trochar was passed into one of the sacs of the tumor, but the contents were so thick that none passed through the canula. The incision in the abdomen was then enlarged, being made to extend to the pit of the stomach. The omentum was found extensively adherent to the superior surface of the tumor, and it was necessary to dissect it carefully loose. One-half of the tumor now protruded through the opening and three of its largest sacs were opened, and their contents evacuated. The mass was then raised from its bed, when it was found to be adherent to about fifteen inches of the small intestines, this was dissected loose, and the tumor turned out through the incision, but left attached by its pedicle, which consisted of the left broad ligament, and the fallopian tube. At this juncture, the patient's pulse failed; her face assumed a death-like aspect, and she gasped as if expiring. By the prompt application of stimulants and excitants she was restored. Her dangerous symptoms were attributed to the removal of the pressure caused by the tumor, and the substitution of chloroform for the ether. The tumor was now raised by Dr. Craig sufficiently to allow Dr. Pierce to pass a needle, armed with a double ligature, through the base of the pedicle; the threads were separated near the needle, tightly secured on each side of the pedicle, and the tumor removed. The ends of the ligatures were secured in the lower angle of the incision. No bleeding vessels were found on examinations, and the ends of the wound was brought together and maintained by means of the interrupted suture and adhesive plaster. Cold water dressings were applied, and a bandage placed round the abdomen, the patient put to bed, and forty drops of laudanum administered. The time occupied in the operation was forty minutes. The intestines caused no trouble during the operation, lying quietly in the position in which they were found when the abdomen was laid open. Two hours after the operation the pulse was one hundred and eight and feeble; the surface cool, and a sensation of sinking experienced. Warm gruel, with one-fourth grain sulph. morphine were prescribed. Six hours after, the patient was more comfortable; morphine repeated. In ten hours, pulse the same; tendency to sleep.

On the 23d at 8 o'clock, A. M., patient comfortable; had several refreshing naps through the night; urine drawn off before day; pulse one hundred and twenty-eight; some appetite; slight hemorrhage from the wound; dressings allowed to remain. At 12 o'clock, M. pulse one hundred and thirty-five; urine has been drawn off twice; abdomen tympanitic; has taken one-fourth gr. sulph. morphia every six hours. At six o'clock P. M., pulse one hundred and forty; abdomen still tympanitic; no pain; tongue coated and brown; slight nausea; prescribed light diet; contiuued morphia.

On the 24th at 12 o'clock, M., pulse one hundred; no thirst or nausea; tongue beginning to clean; considerable appetite; slept well during the night, feels much better; wound healthy; adhesion progressing. From this time forward no unfavorable symptoms occurred.

On the 26th, one-half of the sutures were removed, and adhesive strips substituted.

On the 27th the remaining sutures were removed, and the wound dressed with adhesive plaster and lint. She continued to improve rapidly, and on the fifth week one of the ligatures applied to the pedicle came away, and was soon followed by the other.

Seven weeks after the operation she visited her friends on horseback, and remarked that her health had not been better for a long time. Four months after the operation, she was fleshy and appeared to enjoy perfect health.

The tumor weighed eleven and three-fourth pounds, after its fluid contents were discharged. It was what is called multilocular, and consisted of five large cysts and numerous smaller ones. It appears to have commenced in the left ovary, and a section of it presented numerous fibrous septa, with a highly injected glandular looking substance intervening. The largest cyst appears to have commenced immediately beneath the proper covering of the ovary The contained fluid was viscid, and had floating in it large masses of fat. On microscopic examination, quantities of oil globules were found together with simple isolated cells filled with granules, and large plates of cholesterine crystals. The tumor contained but little blood.

There are several points of interest in this case: 1st, It could not have been relieved by tapping, on account of the viscid nature of the contents. 2d, The rapid recovery of the patient, notwithstanding the adhesions. 3d, The frequent effusions of serum into the peritoneal sac, resulting from the inflammation of this membrane. It may also be interesting to state that the patient menstruated regularly for three or four months, when she became pregnant, and thirteen months after the operation she gave birth to a male child, well formed, and weighing ten pounds at birth, and now, sixteen months after the operation, her health, and that of her fine boy, is good.

*Stanford*, *Aug.* 15, 1855.

